# A Systematic Review of Research on Conformity

**DOI:** 10.5334/irsp.874

**Published:** 2024-07-18

**Authors:** Carla Capuano, Peggy Chekroun

**Affiliations:** 1 Univ. Paris Nanterre, Laboratoire Parisien de Psychologie Sociale – PS2C, FR

**Keywords:** conformity, systematic review, majority influence, social influence, methods, processes, Asch

## Abstract

This systematic review offers a comprehensive overview of conformity research conducted since 2004. Adhering to the PRISMA guidelines, the review identified 48 relevant articles from a substantial pool (literature review conducted between January and April 2023), systematically extracting valuable insights into key findings, methodologies, and future research directions. While recent studies confirm the prevalence of conformity across diverse contexts, echoing Asch’s seminal findings (1951), the review emphasizes the need for a unified understanding of influencing factors, including age, gender, and culture, with contextual variables playing a central role. Advances in digital technology have expanded research possibilities, enabling investigations across diverse digital contexts. Researchers employ innovative methods such as computer-mediated communication (Cinnirella & Green 2007) and virtual reality (Kyrlitsias et al. 2020) to explore conformity within digital spaces that closely mirror real online interactions.

Given the evolving landscape of conformity research, this review advocates for further interdisciplinary and intercultural investigations, comprehensive meta-analyses, and replications to deepen our understanding of this multifaceted phenomenon.

## Introduction

Conformity denotes the process whereby individuals adjust their behavior, opinions, and attitudes to accord with those prevailing among the majority, even in cases where they hold dissenting views (Asch 1956). This phenomenon, initially elucidated by Asch in the 1950s, has since become a focal point of extensive inquiry within the realm of social psychology. Asch employed an original methodology during the 1950s to gauge conformity, employing a visual perception task. In this task, participants were required to verbally identify which of three lines, displayed on the left side of a screen, corresponded in length to a standard line presented on the right side. This visual perception task was characterized by its simplicity and lack of ambiguity. Participants undertook this task alongside individuals who were actually confederates of the experimenter, deliberately providing unanimous incorrect responses on certain trials. The naïve participant would then deliver their response last, enabling observation of the potential influence exerted by this unanimous majority. As of September 18, 2023, the original article by Asch (1951) has garnered 7962 citations on Google Scholar, and Asch’s line judgment paradigm has been replicated numerous times thereafter. Despite individuals occasionally portraying themselves as less conformist than their peers (Pronin et al. 2007), conformity has consistently manifested across diverse contexts and modalities (Bolderdijk et al. 2022; Bond 2005; Cress & Kimmerle 2007; Galinsky et al. 2008; Mori & Arai 2010).

According to the most recent meta-analysis encompassing 125 Asch-type conformity studies, conformity emerges as a robust behavior, exhibiting a weighted average effect size of 0.89 (Bond 2005). Recent investigations have indeed reported conformity rates closely resembling those observed by Asch in the 1950s, exemplified by the replication conducted by Franzen and Mader (2023),^1^ which observed a conformity rate of 33%, mirroring Asch’s rates (1951, 1956). For instance, Goodmon et al. (2020) discovered that 82.67% of their participants conformed to the majority at least once. In Ušto et al.’s (2019) replication of Asch (1956), the conformity rate reached 59.2% (compared to Asch’s 75%). Recent replications and meta-analyses on conformity underscore the robustness of this effect, obviating the necessity to continually assess its existence as it persistently manifests. The processes and factors hypothesized to underlie the phenomenon of conformity are diverse and extensive, and previous research substantiates the pressing need for further inquiry to elucidate definitive explanations regarding this phenomenon.

This systematic review provides an overview of conformity studies conducted since 2004, with the previous literature review focusing on studies concerning conformity and compliance (Cialdini & Goldestein 2004). Furthermore, our investigation indicates a notable absence of systematic reviews addressing conformity to date. The primary objective of this review is to elucidate the latest insights regarding the methodologies employed to investigate conformity and its associated influencing factors. Notably, seven decades have elapsed since Asch’s seminal work on conformity in 1951. Given the societal transformations since those initial studies, it prompts the question: do contemporary individuals exhibit conformity to the same extent as their counterparts 70 years ago? Are behaviors of conformity still as prevalent? Observations on social media platforms suggest a culture that encourages individuals to assert and uphold their opinions, often valuing non-conformity to majority views if in disagreement. This raises the question: have individuals become less inclined towards conformity in reality?

The review is divided into four main sections. The first section outlines the specifics of our PRISMA methodology used for article selection. The next section discusses the methodologies used since 2004, including the various modified paradigms derived from the Asch task. The third section of this review focuses on the results of the included studies and the multitude of factors identified as either influencing conformity or having a negligible impact. Finally, the review concludes with a discussion of the contributions of these recent studies to the field of social influence research, as well as their limitations.

## Method

The method used in this study is based on the PRISMA (Preferred Reporting Items for Systematic Reviews and Meta-Analyses) guidelines: a sequence of 27 steps accompanied by a flowchart (Page et al. 2021). The subsequent section presents the procedures undertaken and the resulting outcomes.

### Eligibility Criteria

In January 2023, we conducted a systematic literature search characterized by a broad inclusion criteria framework. In order to include all pertinent literature, we encompassed not only experimental articles but also literature reviews and meta-analyses.

### Sources of Information and Research

Given the extensive body of research on conformity, spanning several decades, we conducted our investigation using the EBSCOhost platform. Specifically, we selected APA PsycArticles, APA PsycInfo, and APA PsycExtra as primary sources. The search period covered publications from January 2004 to December 2022 (publication date). We employed specific keywords to identify literature on conformity, specifically those relating to Asch’s research. The search query was: “conformi*” in the title AND “Asch” in the title OR “Asch” in the abstract OR “Asch” in the text. This search yielded a total of 1406 articles retrieved from APA PsycArticles (n = 883), APA PsycInfo (n = 479), and APA PsycExtra (n = 44) databases. Following the elimination of duplicates (n = 21), we compiled a final list of 1385 articles.

### Selection of Studies

**Figure 1:**
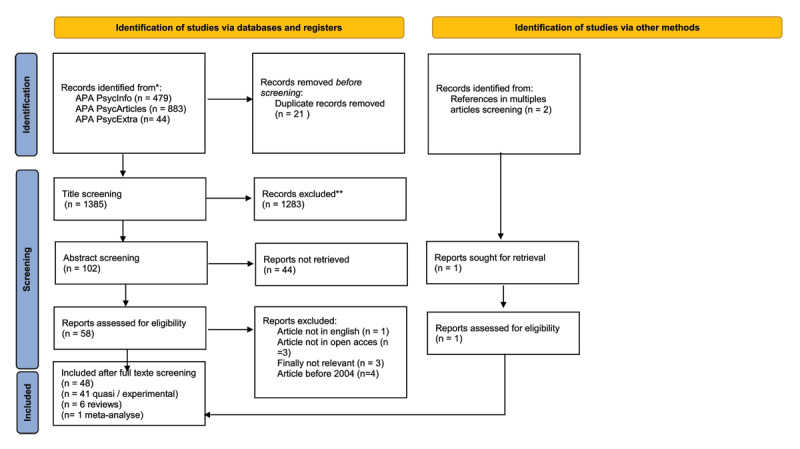
*From:* Page MJ, McKenzie JE, Bossuyt PM, Boutron I, Hoffmann TC, Mulrow CD, et al. The PRISMA 2020 statement: an updated guideline for reporting systematic reviews. BMJ 2021;372:n71. doi: 10.1136/bmj.n71. For more information, visit: http://www.prisma-statement.org/. *Consider, if feasible to do so, reporting the number of records identified from each database or register searched (rather than the total number across all databases/registers).**If automation tools were used, indicate how many records were excluded by a human and how many were excluded by automation tools.

Two independent researchers analyzed the titles and abstracts of 1385 articles. The inclusion criteria for the titles were: any article mentioning conformity or majority influence within the field of psychology (social, developmental, cognitive, clinical). Subsequently, during the abstract screening phase, articles that explicitly measured conformity and/or referenced the utilization of Asch’s paradigm (or any variation) were included. We also considered meta-analyses and literature reviews addressing conformity or majority influence. Following the initial screening of titles and abstracts, 47 and 66 articles were retained by the two reviewers, respectively. A secondary selection process was conducted, ultimately yielding a total of 48 articles for inclusion in this review (41 experimental or quasi-experimental articles, 6 literature reviews, and 1 meta-analysis). Subsequent procedural steps were executed by one of the researchers. A graphical representation of the selection process is presented in Figure 1, following the PRISMA guidelines.

### Data extraction

Given our principal objective of evaluating research on conformity since 2004, we systematically extracted various elements from the selected articles (see Table 1). We developed a comprehensive table including authors’ names, publication dates, pivotal theoretical concepts introduced in the articles, research methods employed, target populations, key findings, as well as limitations and prospective avenues for future research. This comprehensive data extraction process provides a perspective on the extant literature and its advancements, while striving for complete comprehensiveness.

**Table 1:** Methods and main results of the 41 experimental articles included in the systematic review.


AUTHORS	TITLE	DATE OF PUBLICATION	COUNTRY	PARTICIPANTS	METHOD	RESULTS

Griskevicius et al.	Going along versus going alone: When fundamental motives facilitate strategic (non)conformity	2006	USA	Study 1, N = 237 Study 2, N = 69 Study 3, N = 215 (students)	Judging the attractiveness of photos in an online chat room	Self protective mindset induce more conformity in both men and women. The goal to attract a mate reduces conformity in men but increases the likelihood that women will be more conforming.

Cress and Kimmerle	Guidelines and feedback in information exchange: The impact of behavioral anchors and descriptive norms in a social dilemma	2007	Germany	Study 1, N = 111 Study 2, N = 67 (students)	Online task presenting an information exchange dilemma	Participants regulate their behavior by conforming to others, becoming more or less cooperative depending on the salient norm.

Pronin et al.	Alone in a crowd of sheep: Asymmetric perceptions of conformity and their roots in an introspection illusion	2007	USA	Study 1, N = 44 Study 2, N = 40 Study 3, N = 44 Study 4, N = 64 Study 5, N = 92 (students)	Questionnaire on individual conformity perception, scenario reading on a project for university or the purchase of a fashionable item with majority opinion	Individuals perceive themselves less susceptible to influence than their peers Effect of social desirability.

Cinnirella and Green	Does ‘cyber-conformity’ vary cross-culturally? Exploring the effect of culture and communication medium on social conformity	2007	UK	N = 71 (students)	Asch paradigm in face to face or CMC (comunication-mediated by computer)	More conformity in the face to face and CMC conditions than in the control condition. More conformity in the face-to-face condition than in the other two conditions. Effect of culture in face to face condition: participants from individualist country conform less than participants from collectivist.

Galinsky et al.	Power reduces the press of the situation: Implications for creativity, conformity, and dissonance	2008	USA	Study 1, N = 52 Study 2, N = 75 Study 3, N = 45 Study 4, N = 72 (students)	Scenario-based power induction Creative task, tedious task, negotiation task	High power condition participants are more resistant to the majority.

Corriveau and Harris	Preschoolers (sometimes) defer to the majority in making simple perceptual judgments	2010	USA	Study 1, N = 40 Study 2, N = 40 (3–4-year-old preschoolers)	Asch paradigm adapted for children (video confederates)	Majority of children do not conform Negative judgment of adults’ poor video performance.

Pinel et al.	I-sharing and a classic conformity paradigm	2010	USA	N = 44 (women students)	Computer-based interaction Asch paradigm	More conformity in public responses than in private responses. More conformity in non-I-sharing condition than in I-sharing condition.

Mori and Arai	No need to fake it: Reproduction of the Asch experiment without confederates	2010	Japan	N = 26 (students)	Asch paradigm with fMori technique	Almost no conformity in men, rates similar to Asch (1956) for women.

Hanayama and Mori	Conformity of six-year- old children in the Asch experiment without using confederates	2011	Japan	N = 96 (6–7-year-old children)	Asch paradigm with fMori technique	Compared to the results of Mori and Arai (2010), boys conform more than adult males. Girls conform just as much.

Haun and Tomasello	Conformity to Peer Pressure in Preschool Children: Peer Pressure in Preschool Children	2011	Germany	Study 1, N = 96 Study 2, N = 72 (4-year-old preschooler)	Asch paradigm adapted to children (animal images)	Children conform more than a third of trials (study 1 and 2).

Täuber and Sassenberg	Newcomer conformity: How self-construal affects the alignment of cognition and behavior with group goals in novel groups	2012	Germany	N = 157 (students)	Computer-mediated workgroups with positive vs. negative feedback	Conformity is a function of self-image High levels of independent self-construction are associated with low cognitive and behavioral conformity to group goals in cooperative tasks.

Aramovich et al.	Opposing torture: Moral conviction and resistance to majority influence	2012	USA	N = 170 (students)	Online tchat (torture opinion)	More conformity in public responses than in private responses. Influence of majority present in post-test opinions. Moral conviction predicts resistance to majority opinion in public and in private.

Egermann et al.	The influence of social normative and informational feedback on musically induced emotions in an online music listening setting	2013	Germany	N = 5730 (all comers)	Online survey on music evaluations	Participants conform to the assessments of previous participants. Social feedback has more influence on participants’ conformity than informational feedback.

Heerdink et al.	On the social influence of emotions in groups: Interpersonal effects of anger and happiness on conformity versus deviance	2013	Netherlands	Study 1, N = 115 Study 2, N = 73 Study 3, N = 64 Study 4, N = 99 Study 5, N = 86 (students)	Scenario reading and questionnaire	More conformity in cooperative conditions than in competition. Emotions have a social function: anger expressed by the majority leads to a greater feeling of rejection, while joy leads to a greater feeling of acceptance. If an alternative group is possible, then the expression of the majority’s anger encourages individuals to leave the group.

Kundu and Cummins	Morality and conformity: The Asch paradigm applied to moral decisions	2013	USA	N = 33 (students)	Asch paradigm applied to moral dilemmas	Strong conformity effect on moral dilemmas.

Lisciandra et al.	Conformorality A study on group conditioning of normative judgment	2013	Germany	Pre-test, N = 68 Main Study 1, N = 97 (students)	Online questionnaire T1 and T2 (scenario evaluation)	More conformity on social norms and decency than on moral norms. More stable judgment on moral norms between T1 and T2.

Mori et al	Boys, be independent! Conformity development of Japanese children in the asch experiment without using confederates	2014	Japan	N = 20 (7th-grade junior high school pupils, 13–14 years old)	Asch paradigm with fMori technique	More errors from minority participants indicating that they conformed to the majority. Descriptive results show that girls conform more than boys (but no statistically significant results).

Yafai et al.	Social conformity and autism spectrum disorder: A child-friendly take on a classic study	2014	UK	N = 30 (15 autistic children- 15 typically developing children, 2 to 6 years-old)	Asch paradigm adapted to children (Haun & Tomasello, 2011)	Autistic children less compliant than typical children. No difference in performance between children on test trials.

Bos et al.	Reminders of behavioral disinhibition increase public conformity in the Asch paradigm and behavioral affiliation with ingroup members	2015	Netherlands	Study 1, N = 86 Study 2, N = 62 Study 3, N = 60 Study 4, N = 80	Reminders of disinhibition or no disinhibition + Asch Paradigm	In deshinibition condition, participants conform more and have less social distance (more affiliation).

Tu & Fishbach	Words speak louder: Conforming to preferences more than actions	2015	USA	Study 1, N = 66 pairs Study 2, N = 190 pairs Study 3, N = 145 Study 4, N = 150 pairs Study 5, N = 244 Study 6, N = 220	Choice of household products, choice of food, choice of crockery, choice of videos and questionnaire for private vs. public answers with a partner.	More conformity about preferences than about the actions of others.

Beckner et al.	Participants conform to humans but not to humanoid robots in an English past tense formation task	2016	New-Zeland	N = 78 (students native speakers of New Zealand English)	Asch experiment with visual and verbal task (condition robots, human, alone) with public and private responses.	More conformity in the presence of a human than a robot. More conformity if task is ambiguous. Visual task predicts verbal task.

Zhang et al.	Social anxiety, stress type, and conformity among adolescents	2016	China	N = 167 (10–16 year-old adolescents)	Asch paradigm modified with figures and no confederates	Strong conformity observed Interaction between social anxiety and stress type effects on conformity.

Kim et al.	Does children’s moral compass waver under social pressure? Using the conformity paradigm to test preschoolers’ moral and social- conventional judgments	2016	USA	N = 132 (4-year-old children)	Asch paradigm adapted for children	More conformity in social judgment than in moral judgment and visual judgment. No difference in conformity between moral judgment and visual judgment No ethnic difference.

Ng et al	Gratitude facilitates private conformity: A test of the social alignment hypothesis	2016	Singapore	Study 1, N = 212 Study 2, N = 331 (students)	Self-report of a memory Color discrimination questionnaire or shopping task	More conformity when reporting a grateful memory than when reporting joy and neutrality. More conformity observed in study 2 (shopping task) than in study 1 (color discrimination).

Kosloff et al.	Assessing relationships between conformity and meta-traits in an Asch- like paradigm	2017	USA	N = 205 (students)	Asch paradigm with online or face-to-face cartoon evaluation (private and public response)	More conformity in face-to-face condition than in online condition. Stability correlates positively with conformity in face-to-face condition. Positive correlation between Plasticity and Conformity in private evaluation only (no effect in other condiiton).

Botto and Rochat	Sensitivity to the evaluation of others emerges by 24 months	2018	USA	Study 1, N = 49 Study 2, N = 31 Study 3, N = 30 Study 4, N = 34 (14- to 24-month- old)	Robot task for children	Children as young as 24 months perceive public evaluation and are sensitive to information communicated by others.

Cordonier et al	Strong and strategic conformity understanding by 3- and 5-year-old children	2018	USA	Study 1, N = 39 (3 and 5-year-old children) Study 2, N = 17 (5-year-old children) Study 3, N = 31 (3 and 5-year-old children)	Third-person perspective paradigm.	Conformity is used as an affiliation strategy from the age of 5, not before (3-year-olds).

Hellmer et al.	Preschoolers’ conformity (and its motivation) is linked to own and parents’ personalities	2018	Sweden	N = 59 (3 and a half year- old children)	Personnality questionnaire (parents and children) Asch paradigm modified for children	Children of parents who self-identify as extroverts are less likely to conform. Children who were rated as more extroverted by their parents were more likely to conform due to normative reasons, while children rated at a higher level of openness tended to conform for informational reasons.

Ušto et al.	Replication of the ‘Asch effect’ in Bosnia and Herzegovina: Evidence for the moderating role of group similarity in conformity	2019	Bosnia-Herzegovina	N = 76 (political science students	Asch paradigm	Moderator effect of group similiraty (more conformity when majority in-group member (vs. out-group). Replication of Asch result (59% of participants conform at least once).

Kim et al	Using Remote Peers’ Influence to Promote Healthy Food Choices Among Preschoolers	2019	Singapore	N = 89 (3–6-year-old children)	Food choice judgment	Preschoolers conform to healthier food choices endorsed by distant peers.

Ivanchei et al.	Implicit learning in attractiveness evaluation: The role of conformity and analytical processing	2019	Russia	Study 1, N = 88 Study 2, N = 85	Judging the attractiveness of photos	Conformity can be based on a reinforcement learning mechanism and also on unsupervised implicit learning. No difference between learning in a social context and learning without a social context. Implicit learning influences overt responses and also modifies internal criteria for assessing attractiveness.

Pham and Buchsbaum	Children’s use of majority information is influenced by pragmatic inferences and task domain	2020	Canaada	N = 250 (4 and 5 year-old children)	Langage or causal task with majority influence by videos clips	Children’s preference for majority evidence stronger for majority in the linguistic task than in the causal task. Children’s preference stronger when the majority explicitly rejects a choice (rather than in implicit, ignorance or hidden conditions).

Goodmon et al.	The power of the majority: Social conformity in sexual harassment punishment selection	2020	USA	N = 179 (art school students)	Asch paradigm applied to sexual harassment scenarios	82.67% of participants conformed at least once with harsh or lenient punishment choices (incorrect punishment choices) in sexual harassment scenarios.

Kyrlitsias et al.	Social conformity in immersive virtual environments: The impact of agents’ gaze behavior	2020	Cyprus	N = 38	Asch paradigm with virtual reality	Conformity has been observed in an IRV environment. 63.16% of participants conform with agents.

Sibilsky et al	Conformity decreases throughout middle childhood among ni- Vanuatu children: An intracultural comparison	2021	Vanuatu	N = 500 but exploitable 125 (5 to 11-year-old children)	Asch paradigm adapted to children (Haun & Tomasello, 2011)	The older the children, the less they follow the rules (public or private meetings).

Garcia et al.	Normative and informational influence in group decision making: Effects of majority opinion and anonymity on voting behavior and belief change	2021	USA	N = 241 (students)	Jury deliberation task with voting before and after debate	Change in vote is partially mediated by changes in the evaluation of the evidence (regardless of how participants voted in the initial survey).

Schreuter et al.	Trust me on this one: Conforming to conversational assistants	2021	Online survey	N = 163	Online survey with general knowledge quiz	Participants conformed more with the human- voice assistant than with the robotic-voice assistant or the assistant that communicates via text.

Bolderdijk and Cornelissen	How do you know someone’s vegan?” They won’t always tell you. An empirical test of the do- gooder’s dilemma	2022	Netherlands	N = 93 (veg*an all comers)	Asch paradigm applied to food petition (vegan)	If the majority refuses to sign a petition in favour of vegan options, then vegetarian and vegan participants avoid signing. If the experimenter approved the vegan food options, participants did not conform to the majority (and signed the petition).

Qin et al.	Adults still can’t resist: A social robot can induce normative conformity	2022	China	N = 190 (undergraduate and graduate students)	Asch paradigm with 1 robot and 2 confederates	A robot in a minority position can lead to conformity, but less than if it were a human in a minority position.

Sah and Peng	Context-dependent Online Social Influence: Effect of Majority and Minority Comments on Posters and Lurkers	2022	Online survey	Pilot Study 1, N = 35 Pilot Study 2, N = 38 Main Study, N = 354 (321 exploitable) (college students)	Online survey	Participants in posters condition conformed more to the majority and participants in lurkers condition more to the minority opinion. More conformity when the majority is unanimous than when it is unbalanced (regardless of the posting or lurking condition). Moderating effect of group identification and need for cognition on conformity.


## Results

### Developments in Methods for Measuring Conformity

Conformity, initially elucidated by Asch in 1951 through his seminal paradigm involving the comparison of lines to a standard line in the presence of confederates, has undergone methodological refinements and adaptations over time. While Asch’s methodology continues to yield robust results and remains pertinent in contemporary research (Franzen & Mader 2023; Qin et al. 2022; Ušto et al. 2019), innovations have emerged to address practical limitations and better align with evolving societal contexts.

Despite the enduring relevance of Asch’s paradigm, it is constrained by material requirements, particularly the necessity of multiple physical partners. To mitigate this limitation and render the procedure more economical or suitable for diverse populations, researchers have developed alternative paradigms.

Several adaptations have emerged, including the fMORI technique introduced by Mori and Arai (2010). This approach utilizes deceptive eyeglasses to present static stimuli in varied formats. Participants engage in a line discrimination task akin to Asch’s (1956) while wearing special glasses. The glasses worn by the designated minority participant are manipulated to perceive lines of differing lengths from those perceived by the majority. Meanwhile, majority participants wearing unaltered glasses serve as confederates to the experimenter, furnishing discrepant responses to the final participant. This method facilitates the replication of Asch’s experiment without the necessity of recruiting confederates. Study findings suggest that minority group participants, wearing manipulated glasses, provided a greater number of incorrect responses compared to majority participants. It is important to note the limited sample size of the study, comprising only 26 minority participants (10 men and 16 women), with not all confederates included in the analysis (*N* = 78). The reduced sample size may have influenced these results, alongside the heightened task complexity relative to Asch’s original experiment (Mori & Arai 2010). While this technique proves beneficial in circumventing the need for confederates, it necessitates a notably extensive participant pool, as only the responses of the minority participant are considered. Nevertheless, replications are imperative to corroborate the obtained findings and ascertain the validity of the fMORI technique. Hanayama and Mori (2011) conducted a replication of Mori and Arai’s (2010) study with children aged 6–7. Their findings revealed that girls exhibited conformity levels comparable to those of women in the prior study. However, notably, boys demonstrated conformity, contrasting with the absence of conformity observed among adult males in Mori and Arai (2010). Another adaptation of Asch’s paradigm has been utilized to investigate conformity among children as young as four years old. Haun and Tomasello (2011) adapted Asch’s paradigm by replacing lines with animals of various sizes, such as a father, mother, and baby lion, aiming to render the task more engaging and comprehensible for young participants. Furthermore, they devised a back-to-back configuration, eliminating the necessity for human peers, which proves to be a practical approach, particularly when working with children. Each child received a book containing three animals of differing sizes on the left and a control animal on the right. The task entailed identifying whether the control animal corresponded to the father, mother, or baby depicted on the left. One child received a different book from the rest, akin to Mori and Arai’s (2010) experiment employing manipulated glasses, thereby serving as the minority participant. Haun and Tomasello (2011) yielded findings akin to those of Mori and Arai (2010) in children. Their modified paradigm successfully evoked conformity in 18 out of 24 minority children, who provided more incorrect responses compared to their peers. The methodology employed in this study, tailored for children, shares a common limitation with the fMORI technique, necessitating a large sample with only minority children’s results being included in analyses. However, this limitation persists even with a majority physically present alongside the participant. To mitigate these challenges, online procedures have been developed.

#### Online Conformity

The internet has opened up novel modes of communication, facilitating individual participation in social networks, forums, and web platforms. These digital environments offer fertile terrain for investigating social influence. The advent of these novel communication channels has prompted inquiries into the influence of majority opinion within such domains. Is conformity discernible in online settings? Do conformity rates vary across different online environments? Consequently, methodologies for assessing conformity have evolved in tandem with technological progress over the past two decades. Advances in technology have made it possible to overcome some of the limitations inherent in the previous methods.

Cinnirella and Green (2007) investigated conformity utilizing computer-mediated communication (CMC). The study replicated Asch’s stick discrimination experiment under two conditions: face-to-face (similar to Asch’s original study) and completely online CMC. Participants were led to believe they were concurrently performing the task with others, whereas in reality, they completed it individually, with computer-programmed responses. Each participant sequentially indicated their answer, with the naive participant consistently responding last. Results indicated participants conformed to majority influence in both face-to-face and CMC conditions, albeit with significantly lower conformity rates observed in the CMC condition. In alignment with this investigation, Aramovich et al. (2012) employed a CMC approach to explore the influence of the majority on participants’ moral beliefs. Despite morality’s significance to individuals, findings revealed that 80% of participants expressed reduced opposition to torture compared to their initial declarations in the pre-test, indicative of majority influence. To further investigate majority influence in a digital environment, Kyrlitsias et al. (2020) replicated Asch’s study using virtual reality. They also noted that their participants conformed to the virtual agents, highlighting that their high conformity rate (63.16% compared to Asch’s result of 75%) could be elucidated by various factors, including the level of immersion or anonymity.

Depending on the research question, there are a variety of methods for studying conformity (Garcia et al. 2021; Ivanchei et al. 2019; Pinel et al. 2010; Sah & Peng 2022; Täuber & Sassenberg, 2012). The emergence of these novel methods allows for the study of conformity without an experimenter, the adaptation of procedures to the population being studied (e.g., children), or the study of conformity in online environments. These methods raise questions about the emergence of novel conformity. Are the factors influencing susceptibility to conformity before the 2000s comparable in online environments? Do motivations for conformity remain consistent across contexts? Moreover, these new methodologies have revealed that conformity rates in online environments parallel those observed in traditional Asch paradigm studies with human confederates, underscoring the persistent nature of conformity even in virtual environments. Future research could address the impact of AI and virtual reality on individuals’ online behavior, as their growing prevalence requires new investigations. The rapid integration of new technologies into everyday life, exemplified by the increasing ubiquity of AI (such as ChatGPT) and virtual reality headsets, presents methodological opportunities for researchers to deepen their understanding of the factors contributing to conformity, an area where consensus remains limited.

#### Development of a New Conformity Scale

While the primary approach to measuring conformity typically entails creating situations of majority influence through exposure to an influencing source or employing confederates or fictitious majorities, some researchers have adopted scales to assess the degree of conformity, treating it as a trait. This method effectively simulates real-life scenarios and directly gauges individuals’ conforming (versus non-conforming) responses. Brügger et al. (2019) propose a 33-item conformity measurement scale aimed at evaluating variations in individuals’ conformity levels. This innovative scale seeks to address limitations associated with existing scales (Comrey 1970; Mehrabian & Stefl 1995; Schwartz 1992), which rely on evaluative statements or introspective self-reflection items that may inadvertently introduce measurement error or social desirability bias. The authors of this scale assert several advantages. First, it is based on only two parameters: the individual’s level of conformity and behavioral difficulty. Moreover, it assesses past activities rather than relying on self-evaluations, thereby mitigating methodological challenges associated with evaluating abstract concepts (Brügger et al. 2019). Furthermore, the Campbell paradigm employed in developing the scale ensures its psychometric robustness. Nevertheless, Brügger et al. (2019) acknowledge certain limitations of the scale. Firstly, it was calibrated on a sample that predominantly comprised individuals with higher levels of education compared to a representative sample of the general population. Similarly, the perceived difficulty of the items may have varied across demographic groups, leading the researchers to suggest that further testing is needed to determine the adaptability of the scale to diverse populations. In addition, they suggest that the reliability of the Rasch separation should be improved to better distinguish between medium and high levels of conformity, thus providing more reliable results. While Brügger et al.’s (2019) scale holds promise as an effective tool for measuring conformity, as posited by the authors, its validity and reliability necessitate confirmation through future research. Additionally, it could be explored as a moderator, but this requires further investigation.

In this section, we have examined the methods employed for observing and measuring individual conformity since 2004. Technological advancements have facilitated overcoming some of the costly limitations of the initial task. However, they have also introduced new challenges, such as the potential exclusion of a significant portion of the sample or the physical absence of the majority, potentially diminishing normative influence. Nonetheless, these adapted paradigms afford researchers the flexibility to choose the most suitable procedure for their specific objectives, technical capabilities, and target population, thereby constituting a notable advantage.

### Is There a (Non)Conformist Profile?

#### Age and Gender

Since Asch’s seminal studies in the 1950s, researchers have endeavored to elucidate the factors underlying individual conformity by exploring personality traits and inter-individual differences. However, no definitive effect of traits has been established thus far. Consequently, scholars have persisted in their investigation of this question. These factors are theorized to influence individuals’ susceptibility to conform to majority opinions.

Recent studies on conformity have revealed diverse effects. This section examines the complex interactions between factors that contribute to susceptibility to conform. Asch’s (1956) study concentrated on a sample comprising 123 men aged between 17 and 25. Subsequent studies have explored age and gender as potential moderators of conformity. Consistent with prior research findings, most recent studies suggest that conformity is a behavior exhibited by both males and females, with no significant disparity in conformity scores (Bos et al. 2015; Garcia et al. 2021; Hanayama & Mori 2011; Haun & Tomasello 2011; Hellmer et al. 2018; Kim et al. 2016; Lisciandra et al. 2013; Schreuter et al. 2021; Ušto et al. 2019). However, some studies have indicated potential gender differences in conformity, suggesting that women may be more inclined to conform than men. For instance, Sibilsky et al. (2021) conducted a study on conformity rates among children aged 5 to 11 (*N* = 125, 59 boys) across eight communities in Vanuatu. Employing Haun and Tomasello’s (2011) procedure adapted for children, they observed that girls exhibited higher levels of conformity compared to boys. Moreover, it was observed that conformity decreased as boys aged, whereas girls’ conformity remained relatively stable across different age groups. Additionally, Griskevicius and colleagues (2006) conducted a study aiming to elucidate the gender disparity in majority influence by investigating the impacts of two primary motives: partner attraction and self-protection. The study employed a method involving the priming of self-protection versus partner attraction motives through imaginative scenarios. Participants were asked to evaluate a painting (Study 1), responding to subjective versus objective questions (Study 2), or answering subjective versus objective questions with unanimous majority versus split opinions (Study 3). The discrepancy in responses before and after being influenced by majority ratings was utilized to evaluate participants’ conformity levels. Their findings suggest that men are less inclined to conform when endeavoring to attract a partner, while women exhibit higher levels of conformity in this context. No gender differences were observed when the focus was on the self-protection goal. This set of results suggest that motivations for conformity among men and women may evolve over time, potentially accounting for these findings. Similar findings were reported by Zhang et al. (2016) in a sample comprising 152 adolescents aged 10 to 16, utilizing a modified Asch task featuring figures. Mori et al. (2014) also documented analogous results among both 13–14-year-old adolescents and undergraduates (Mori & Arai 2010). These findings contrast with those observed in children aged 6–7 (Hanayama & Mori 2011). Upon comparison of these studies, it becomes evident that only a minority of them demonstrate a gender effect, and gender is not consistently considered as a potential moderator of conformity across the majority of studies examined (out of 78 studies reviewed, 64 did not mention gender). The findings suggest that conformity is a behavior observable from an early age (Haun & Tomasello 2011; Pham & Buchsbaum 2020; Yafai et al. 2014). Botto and Rochat (2018) demonstrated that children as young as two years old were sensitive to the evaluations of others and could adapt their behavior based on the attention and feedback received from the experimenter. This sensitivity increased with age, as children gradually comprehended the significance of conforming to their peers’ expectations for social acceptance. Kim et al. (2019) observed a positive correlation between age and conformity rates among children aged 3–6 years in their food preferences for vegetables, noting that older children exhibited greater conformity than younger ones. Corriveau and Harris (2010) found that children aged 3–4 years displayed lower conformity rates (20% in Study 1 and 26% in Study 2) compared to adults in Asch’s experiment (33%). Hence, as children mature, they come to understand the importance of conforming to their peers’ expectations for social acceptance. According to Cordonier et al. (2018), children as young as five years old begin to grasp the significance of adhering to peer expectations for social acceptance, a developmental milestone that younger children have yet to attain. This fosters the acceptance of conformity as a social strategy. As children progress in age, they may employ conformity as a means to avoid social exclusion, becoming more inclined to conform to their peers’ choices as they grow. However, the strength of this positive correlation between age and conformity largely depends on the specific object chosen to study conformity.

Contradictory results observed in other studies suggest that children may become less likely to conform as they grow older. However, it should be noted that these conclusions were drawn from various types of inter-group comparisons. No longitudinal study has demonstrated that individuals develop resistance to majority influence throughout their lives. Kim et al. (2016) examined the conformity of preschool children, approximately three years old, using moral, social-conventional, and visual perception tasks adapted for children (Corriveau & Harris 2010). The study presented four moral and four social transgressions to the child on a computer. A video featuring the response of the majority, consisting of two children of the same age as the participants, was shown to the child. Subsequently, the experimenter in the video asked the child whether it is acceptable to transgress the convention, whether moral or social. The results indicated that older children were less likely to conform with moral conventions (e.g., hitting another child and shoving another child) and the visual perception task than with social conventions (e.g., a boy wearing nail polish). The researchers suggest that this discrepancy may be attributed to the nature of the studied objects. Moral conventions become integrated into an individual’s value system during development and tend to remain stable over time. According to Kim et al. (2016), transgressing moral conventions typically entails more severe consequences for others compared to transgressing social conventions, which are characterized by greater flexibility and variability in norms. As children develop, they acquire an understanding of the differing importance of norms based on their nature (moral versus social), potentially accounting for their decreasing likelihood to conform as they mature. Children grasp that conformity is valued in situations governed by social norms. Conversely, regarding moral norms, children understand that conformity involves adhering to behaviors that may have negative repercussions, such as social sanctions or exclusion from the group. Similar findings were reported regarding a visual perception task in the study conducted by Sibilsky et al. (2021) among children aged 5 to 11.

Recent studies investigating the influence of age and gender on conformity have failed to identify significant effects attributable to these variables. These recent findings have not presented any novel insights that contradict previous conclusions. A meta-analysis of the most recent studies could be conducted to substantiate the diversity of effects associated with these variables.

#### Culture and Conformity

Similar to gender or age, the demographic specificity of Asch’s study, which concentrated on a population of Western men in the 1950s, prompted researchers to explore the influence of culture on conformity. Conformity has been investigated across various countries to determine the extent to which this behavior can be generalized across different cultures.

Of the 45 experimental articles selected for this review, studies were conducted across diverse countries, including Germany, Switzerland, England, Bosnia-Herzegovina, Canada, China, Japan, Singapore, the United States, and Vanuatu. Although most studies do not explicitly mention a cultural effect, some interpret their findings in terms of cultural factors, such as tendencies towards individualism or collectivism. Bond and Smith (1996) conducted the latest meta-analysis of the impact of culture on studies utilizing Asch’s paradigm (1952b, 1956). It included 133 studies from 17 different countries and was particularly interested in the cultural values of individualism and collectivism (between-culture level), measured in these studies by three different scales (Hofstede 1983; Schwartz 1994; Trompenaars 1993). Their results showed that conformity appears to be higher in collectivist cultures than in so-called individualist cultures. More importantly, the impact of these cultural values was greater than other situational variables considered key moderators, such as majority size (Bond & Smith 1996). However, conclusions regarding the link between culture and conformity depend on whether the study is conducted at an intercultural or intracultural level. For instance, Tu and Fischbach (2015) investigated the intercultural level by replicating one of their studies in China (n = 84), Korea (n = 102), and the United States (n = 57). The study demonstrated that there was no difference in conformity among the three samples, suggesting that cross-cultural factors do not significantly influence conformity. These findings contradict those of Bond and Smith (1996), but could be explained by the disproportionate or overly homogeneous nature of the samples, such as a predominantly student population. The comparison drawn from the study is limited in its ability to draw conclusions on the effect of culture. This limitation also arises from the fact that in most of the studies (Cinnirella & Green 2007; Corriveau & Harris 2010; Kim et al. 2016), only the socio-demographic variable of the country of birth and/or residence was measured, and no other cultural variables were considered. Consequently, certain dimensions of culture suggested by Triandis (1996) remain unexplored in these studies. These dimensions include *tightness*, which pertains to deviations from norms and the sanctions/punishments that may ensue, and cultural complexity, which encompasses the multitude of cultural elements differing from one culture to another, such as religious, political, or social norms. Furthermore, the studies do not address the importance of hierarchy in groups, as defined by the terms Vertical and Horizontal Relationships (Triandis 1996). This hierarchy can be critical in elucidating certain social behaviors in specific countries. Consequently, the literature fails to examine the significance of adhering to norms and tolerating deviations from these norms. It could be posited that in cultures where *tightness* is valued, conformity would be more emphasized. Culture encompasses more complexity than merely the collectivist versus individualist dimension, and future studies must explore these other dimensions. This restricted conclusion on the effect of culture, often reduced to a demographic variable of the country of residence, may distort the overall conclusions on the subject. To effectively conclude on the effect of culture on conformity, further research is warranted. It is noteworthy that many tools exist in order to measure various cultural dimensions. However, the use of multiple measures can be a source of confusion between the concepts (Taras et al. 2009). Therefore, for future studies to yield more robust conclusions regarding the impact of culture, it is imperative to validate and employ diverse measures to ensure accurate assessment of the dimension(s) of interest. This necessitates the utilization of large, representative samples from the population, which can pose financial and logistical challenges for researchers. The hypotheses could be tested through the development of multi-site distributive studies. Nonetheless, such studies are indispensable for attaining a deeper comprehension of the phenomenon.

#### Personality Traits

While age, gender, and culture have been explored as factors influencing susceptibility to conformity, the consideration of personality variables in this context remains an intriguing avenue for exploration. Kosloff et al. (2017) conducted an examination of these matters by investigating the correlation between two distinct dimensions of the Big Five personality traits, as measured by the NEO Five Factor Inventory-3 (McCrae & Costa 2010): Stability (reversed neuroticism, higher agreeableness, and conscientiousness) and Plasticity (openness and extroversion), and rates of conformity in women. Their findings revealed a positive correlation between Stability—portrayed as a potential precursor to adherence to social norms—and conformist behavior. Conversely, they found no association between Plasticity and conformity (Franzen & Mader 2023).^2^

These findings align with those of Hellmer et al. (2018), who explored the influence of personality traits on 3.5-year-old children (n = 59). The parents of the children first completed online questionnaires to assess their own and their child’s personalities. Next, the child completed an age-appropriate version of the Asch (1956) task in a laboratory setting. The task involved watching a video and determining which of two animals had the most dots. While watching the video, the child observed four adults (2 female and 2 male) fail to answer some of the questions. The researchers used an eye-tracking tool to observe the participants’ private responses and distinguish between two motivations to conform. If a child gave an explicit response that was incorrect, but their hidden belief was correct (i.e., looking in direction of correct answer), the researchers considered that the child had conformed normatively. When a child provided an incorrect response that aligned with their incorrect hidden belief (i.e., looking at the wrong answer), researchers labeled this as informational motivation. The study found that children of parents who self-identify as extroverts are less likely to conform to incorrect responses shown in the video. Furthermore, children rated as more extroverted by their parents were more likely to conform due to normative reasons, while those rated at higher levels of openness tended to conform for informational reasons. Current research has still not definitively and consistently demonstrated the impact of personality traits on conformity, indicating that further studies are required.

Overall, these results suggest that conformity is a complex behavior observable across various ages and irrespective of participants’ gender, culture, or personality traits. Longitudinal studies tracking changes in conformity across age and gender could offer valuable insights. Moreover, future research could systematically explore the gender effect, facilitating meta-analytical investigations on the topic. More comprehensive research on culture is warranted, avoiding simplistic comparisons, to yield more robust conclusions. However, based on the studies reviewed herein, individual factors seem to have limited moderating effects on conformity. The study by Kosloff et al. (2017) shows an effect of Stability on conformity (*r* = .34), and Hellmer et al. (2018) suggests an effect of openness and extraversion in children, but the results for other personality traits are inconclusive. Recent research affirms the findings of studies conducted in the latter half of the 20th century, indicating that conformity is predominantly influenced by external factors. This aspect will be further discussed in the subsequent section.

### Motivations to Conform

As elaborated in the preceding section, a comparative analysis of recent research findings on conformity indicates that it is a behavior evident in both men and women, irrespective of age or cultural background. In the following section, we will examine how conformity is primarily shaped by the motivations of individuals, depending on the stimuli used to assess conformity. The challenge of reaching definitive conclusions about conformity stems in part from the diversity of observed behaviors and the existence of different processes that lead to conformity. Building upon Asch’s seminal work (1951), Deutsch and Gerard (1955) suggested two explanatory processes for the phenomenon of conformity: normative influence, wherein individuals conform in response to social pressure, and informational influence, wherein individuals conform due to cognitive uncertainty. These processes are driven by two primary motivations: the desire for social acceptance and the pursuit of accuracy (Cialdini & Goldstein 2004). Asch’s seminal research on conformity commenced with a simple and unequivocal visual perception task. The study found that individuals often conformed to the majority opinion, even when they held dissenting views and were aware of the correctness of their own response. Various external factors have been identified as significant determinants of conformity, including the size of the majority and the nature of the task (Bond & Smith 1996). Recent investigations have demonstrated that conformity levels fluctuate depending on the stimuli under examination or the characteristics of the majority. This implies that external influences predominantly shape individuals’ levels of conformity, and that individuals exhibit varying motivations to conform to different extents based on these influences.

#### The Power of Norms

The impact of majority pressure has frequently been examined in contexts involving matters of minimal personal value or significance to individuals, such as visual perception tasks akin to Asch’s 1951 study or expressing opinions on musical choices (Egermann et al. 2013). The findings of such studies often lead to superficial and transient changes in opinions or behaviors, referred to as public conformity (Deutsch & Gérard 1955). Researchers have endeavored to ascertain whether majority influence extends to attitudes, opinions, or deeply ingrained norms within individuals’ value systems, such as moral beliefs. Preliminary findings suggest that the inclination to conform diminishes in relation to the values or norms at stake.

One type of norms investigated for susceptibility to conformity are moral norms. Moral norms tend to remain relatively stable over time and are internalized from an early age, encompassing principles such as refraining from stealing or being violent towards others (Kim et al. 2016). In a task involving moral dilemmas, Kundu and Cummins (2013) demonstrated that individuals conformed to the majority opinion. Goodmon et al. (2020) asked participants to assess the appropriateness of three different sanctions in a sexual harassment scenario, revealing an overall agreement rate of 46% for sanctions considered inappropriate. However, recent research has supported the conclusion that moral norms are more resistant to transgression than others. Consequently, it would be more challenging for individuals to comply with a majority that contradicts their moral values. Aramovich et al. (2012) conducted a study focusing on attitudes toward supporting or opposing torture, both before and after individuals were exposed to a majority viewpoint facilitated through an online chat platform. Their findings indicated that the level of moral conviction serves as a predictive variable for resistance to the majority and, consequently, the degree of conformity. Individuals with higher levels of moral conviction demonstrate greater resistance to the majority (i.e., lower conformity). Lisciandra et al. (2013) conducted a normative judgment procedure on scenarios involving violations of moral, social, and decency norms to investigate differences in susceptibility to conform to these types of norms. The study found that individuals are more likely to conform to the majority on social norms and conventions than on moral norms. Similar results were observed in four-year-olds (Kim et al. 2016), who tended to conform less to moral issues (e.g., teasing another child and calling another child names) than to issues related to social conventions (e.g., wearing a bathing suit to day care, standing during story time). Individuals perceive fewer inter-individual benefits of conforming if it involves transgressing a moral norm, unlike in other contexts such as social norms. Researchers agree that transgressing moral standards has more negative consequences on relationships with others and the group. While these findings indicate that conformity can indeed extend to moral issues, most studies imply that conformity to moral norms is less pronounced than conformity to social norms. The significance of moral values subject to group influence emerges as a moderating factor in the manifestation of conformity. These results suggest that norms are powerful factors influencing conformity. The aim of future studies will be to determine whether these norms have more weight than other factors such as the size of the majority or the anonymity of responses in susceptibility to conform. Future research on the impact of standards will investigate the effects of different standards and assess the extent of conformity based on these variations. Indeed, the multiplicity of factors at play in situations of social pressure makes it difficult to draw conclusions about the weight of particular factors (e.g., individual vs. situational). The normative nature of conformity can also be explored. Is it the norm to conform in one situation or another? Are there differences in the perception of normativity between individuals or groups?

#### Who Is the Majority?

In addition to norms, the characteristics of the majority group, which serve as a source of influence, may also have an effect on the rate of conformity. In this section, we will discuss recent findings that have highlighted specific group attributes that have the potential to increase or decrease control over individuals, thus acting as moderators of conformity.

As early as 1958, Kelman highlighted the different ways in which the majority exerted pressure on individuals to conform. More precisely, he identified three levels of conformism resulting from these different forms of influence, which themselves depend on certain characteristics of the majority group. When the majority has control over the means and individuals are under its control, individuals will conform with the aim of obtaining a favorable response from the group or avoiding social sanctions, a type of influence that Kelman (1958) calls compliance. On the other hand, if the majority is attractive and the relationship between individuals and the group is made salient, then individuals will conform to maintain a satisfactory relationship by identifying with the majority and adopting its point of view in a process called identification. A third level of conformism (known as internalization) is linked to the influence exerted by a majority perceived as a credible and relevant source, with a behavior consistent with the individual’s value system. These three forms of conformity described by Kelman clearly show that the characteristics of the majority and the relationships the individual has with it lead to changes in conformity.

The characteristics of the majority group, such as group size or the distinction between out-group and in-group members, have been extensively studied to determine their potential influence on conformity (Bond 2005). Recently, Ušto and colleagues (2019) replicated Asch’s (1956) experiment in Bosnia-Herzegovina and demonstrated that individuals conform more to in-group members than to out-group members. Specifically, individuals were more inclined to conform to in-group members when their group identity was prominently highlighted. Empirical studies indicate that the stronger individuals identify with the majority, feeling akin or closely connected to this majority (e.g., friends, family), the more inclined they are to conform (Tu & Fishbach 2015; Ušto et al. 2019). This phenomenon is notably congruent with the theoretical principles of social identity theory (Tajfel 1982), wherein maintaining a positive group image necessitates alignment with in-group members. Additionally, according to self-categorization theory (Turner 1991), agreement with in-group members serves as a marker of subjective validity, indicative of shared norms. Thus, individuals are more inclined to share a social reality with in-group members and, consequently, foster a greater propensity for conformity (Ušto et al. 2019). Identification with the group emerges as a pivotal determinant of conformity pressure: the less salient or important the group is, the less likely conformity becomes. This attenuation in conformity underscores the centrality of group identification as a potent moderator, highlighting that individuals are less susceptible to influence exerted by a majority with which they lack identification. Moreover, the signals sent by this majority are real clues guiding individuals to adapt their behavior in line with it. Previous studies have primarily examined the emotions experienced by individuals under the influence of the majority and motivated to conform. The emotions conveyed by members of the majority toward an individual subject to influence have garnered considerable attention in the study of conformity, notably emphasized by Heerdink et al. (2013). These emotions are considered indicators that enable individuals to assess the alignment of their behavior with prevailing situational norms, thereby influencing the extent of their conformity. When the majority expresses anger, individuals tend to experience heightened feelings of rejection by the group and are consequently more likely to conform and affiliate with the group (Heerdink et al. 2013). This effect is amplified when the group is less familiar to the individual. Moreover, in instances when the group has a cooperative objective and expresses anger directed toward the minority, individuals are more inclined to conform (Heerdink et al. 2013). Conversely, the expression of positive emotions by the majority carries distinct implications for conformity dynamics. The majority’s expression of joy fosters a heightened sense of acceptance within individuals, serving as an implicit signal that their behavior aligns with prevailing group norms, thereby encouraging further conformity. On the individual side, experiencing positive emotions such as gratitude or joy when confronted with a dissenting majority promotes conformity, especially in private contexts. In this specific context, the experience of gratitude fortifies social bonds with others, thereby contributing positively to an individual’s social integration within the group (Ng et al. 2017).

The results of studies into the effect of non-human peers (robots, virtual assistants, etc.) on conformity are consistent with the above conclusions. From chatbots on online shopping platforms to conversational assistants, technology is increasingly integrated into daily life. To what extent do individuals allow themselves to be influenced by these robots? Over the first half of the 21st century, research sought answers to this question, revealing that robots wield weaker influence over individuals compared to their human peers. A study by Beckner et al. (2016) involved exposing individuals to a majority viewpoint espoused by robots in a task reminiscent of Asch’s conformity experiments. The results showed that this robotic majority exerted no influence on the responses of participants, who steadfastly maintained their independence. These findings align with a study by Schreuter et al. (2021), demonstrating that people are more amenable to the human voice of a conversational assistant compared to that of a robot. Given the evolution of artificial intelligence (AI) and the increasing humanoid features of technological assistants—such as voice and language capabilities—it is plausible that these entities may emerge as more potent sources of conformity in the future. Consequently, future research exploring the dynamics between individuals and AI holds considerable promise in discerning their impact.

Recent studies on conformity indicate that external factors, such as the type of standards or the relationship with the majority group (in-group vs. out-group), strongly influence conformity. Manipulating these factors could lead to more or less conformity. Future studies could investigate whether varying characteristics of the majority promote different levels of conformity. This could raise significant questions regarding the promotion of pro-social behaviors such as eco-friendly behavior, healthier eating, sustainable consumption, or adopting a healthy lifestyle, which need to be addressed in studies on majority influence.

### Are There New Theoretical Explanations to Conformity?

Over the past few decades, research on conformity has aimed to understand the relationship between various factors, such as majority size or response type, and conformity. In his meta-analysis, Bond (2005) suggests that the multitude of variables involved in conformity makes it challenging to isolate some of these variables and infer their moderating role. In particular, he concludes that future research should examine the different motivations that lead to conformity, as well as how the characteristics of the task or context can lead to different motivations (Bond 2005).

Asch’s (1951) seminal work has been widely replicated; however, despite the robustness of conformity as a phenomenon, there are ongoing debates, even in more recent research, about the interpretation of his findings. In their review, Spillane and Jouillié (2022) refer to Friedrich’s theory of authority (Friedrich 1958) to explain Asch’s findings. The observed conformist behavior in laboratory settings results from participants’ perception of the experimenter’s authority. Participants conform to the expectations of this authority within the specific confines of a laboratory setting, without necessarily displaying similar behavior in ‘real life’ situations.

Conversely, Hodges and Geyer (2006) argue that Asch’s experiments demonstrate that individuals do conform, but not consistently, and instead seek consensus. Consequently, conformity rates never exceed 75% for ‘typical’ participants (i.e., excluding those who consistently conform or never conform). According to pragmatic value theory (Hodges & Geyer 2006), Asch’s scenarios represent dilemmas in which individuals balance between truth (implying nonconformity) and consensus (implying conformity). Thus, instances of conformity reflect the individuals’ desire to fit in with the group, whereas maintaining one’s position is an expression of disagreement. This theory suggests that individuals are more likely to conform in the presence of strangers than among friends because it is more difficult and less straightforward to express dissent when dealing with unfamiliar individuals, where trust and honesty have not yet been established (Hodges & Geyer 2006). In contrast, theories of conformity (e.g., Cialdini & Trost 1998; Graham 1962), as well as research on the effects of group formation discussed above, anticipate the opposite trend: increased identification and cohesion are expected to exert stronger normative and informational pressures. These theories agree that conformity is a socially motivated behavior, either to acquiesce to the authority of an experimenter or to conform to group norms. Furthermore, Cialdini and Goldstein (2004) suggest in their literature review, that conformity serves three fundamental goals: accuracy, belonging, and maintaining a positive self-image. These goals are aligned with the theoretical considerations mentioned above. Individuals conform to satisfy their need for group affiliation, avoid appearing deviant and thereby promote a positive self-image, and to respond appropriately to group-derived information (i.e., accuracy). These theoretical propositions are broadly consistent with the initial concepts of Deutsch and Gerard (1955) or Kelman (1958), who distinguished between social and cognitive motivations supporting two pathways to conformity: informational influence and normative influence. Over the years, research has primarily focused on understanding the underlying processes of conformity, with less emphasis on interpreting results related to individual independence. Griggs (2015) investigated the prevalence of mentions of nonconformity results in 30 introductory psychology and social psychology textbooks, and found that only 15% of the selected textbooks referred to the proportion of independent responses (i.e., nonconformity). This finding highlights the authors’ preoccupation with conformist responses, leaving the exploration of non-conformity as an avenue for future research.

#### Neurocognitive Mechanisms

The question of why individuals conform has also captured the interest of researchers in the field of neuroscience. In particular, they seek to understand the neurological processes that occur when individuals are subject to the influence of the majority. Advances in imaging technologies have facilitated progress in understanding the neural basis of conformity. Various imaging techniques such as functional magnetic resonance imaging (fMRI), event-related potentials (ERP), or even transcranial magnetic stimulation (TMS) have been used to study the neurocognitive mechanisms underlying conformity (see Schnuerch & Gibbons 2014). These studies show that brain regions such as the posterior medial frontal cortex, which is involved in error detection and the need for behavioral adaptation, and the ventral striatum, which is responsible for the regulation of motivation and impulses, are activated in situations that induce conformity. These findings suggest that conformity is primarily based on an error-based reinforcement learning mechanism (Schnuerch & Gibbons 2014). Specifically, when individuals experience conflict between their judgments and those of the majority, this conflict generates an error or even a reward/punishment signal. Furthermore, these studies have shown that such conflict can induce negative affect, which conformity serves to mitigate. Previous research has linked activation of the dorsal medial prefrontal cortex (dmPFC), a region responsible for processing fear and anxiety-related information, to cognitive dissonance (Izuma et al. 2010). Thus, during conflict with a majority group, individuals may experience cognitive inconsistency, which they alleviate by conforming. These findings shed light on the neural processes involved in conformity and provide insights into the associated affective, social, and cognitive conflict.

From an evolutionary perspective on comparative cognition, Claidière and Whiten (2012) examine conformity in humans compared to animals. It appears that animals such as rats or fish can exhibit ‘conformist’ behaviors in foraging strategies. When learning new skills, chimpanzees tend to choose one behavior by imitating their peers when given a choice between two behaviors. Similarly, sparrows choose to emit the sound most commonly used by other birds. These studies suggest that conformity can be observed in animals and that the behaviors demonstrated by conspecifics serve as an informational basis for individuals to guide their own conduct. However, this conclusion remains interpretative, and there is no empirical support for similar influence processes in humans.

These findings provide insight into how the brain processes information received from the majority and how conformity plays a role in this process. Recent neuroscientific studies have provided new insights into the mechanisms of conformity and support the experimental findings of previous studies.

## Discussion

The range of articles examined in this review underscores that conformity remains a domain of research yet to be exhaustively explored. This behavioral phenomenon is multifaceted, influenced by a multitude of factors, each with varying degrees of significance in shaping susceptibility to conformity. Moreover, the influence of these factors fluctuates contingent upon the subject under investigation, the demographic characteristics of the population, and the methodological approaches adopted. Studies that examine conformity suffer from several limitations, predominantly stemming from methodological constraints.

To experimentally investigate conformity, the utilization of confederates to induce majority influence was imperative in the initial studies on this subject. The recruitment of confederates presents challenges for researchers, both financially and logistically. Consistency is crucial as the same confederates must be employed for all participants and they must be adequately trained to provide incorrect responses at the designated junctures. In response to these challenges, researchers have leveraged technological advancements in recent decades to develop new online methodologies, circumventing the need for confederates. While online procedures are gaining traction among researchers, they entail notable limitations when examining conformity. Firstly, the absence of physical presence in online settings may diminish the impact of majority influence. This visual anonymity may attenuate feelings of accountability and anxiety associated with evaluation, resulting in reduced levels of conformity compared to traditional Asch-type tasks (Cinnirella & Green 2007). Additionally, the design of online chat platforms varies across studies, potentially influencing perceptions of majority influence. Thus, it is imperative to replicate studies utilizing these novel online methodologies, such as computer-mediated communication (CMC) or online chat, to ensure the reliability and specificity of findings. Furthermore, as evidenced in this review, conformity has been explored across a spectrum of topics including moral dilemmas, visual tasks, and problem-solving tasks. Despite the diversity in the objects of study and task types, a notable takeaway from this review is the resilience of conformity as a behavioral phenomenon.

The proliferation of new technologies since the 2000s has facilitated the expansion of social networks, online chat platforms, forums, and other virtual spaces. Consequently, individuals now dedicate a significant portion of their time to these digital environments, prompting inquiries into the social influences to which they are exposed. As individuals become increasingly and readily exposed to the opinions and attitudes of a vast number of peers, it becomes crucial to evaluate the ramifications of this continual exposure on their own beliefs and values. Does this heightened exposure lead to greater conformity among individuals? Do they integrate the information disseminated by the majority into their personal value systems? Can such conformity precipitate genuine changes in individual behavior? The examination of online conformity necessitates contextualization within the broader societal issues prevalent in these digital environments, including online harassment, misinformation dissemination, and radicalization. Therefore, future studies should explore these multifaceted issues in greater depth to elucidate their complexities and implications.

In addition to methodological limitations, studies often explore cultural differences as potential moderators of conformity, particularly regarding the collectivist-individualist dimensions. However, conclusions regarding the impact of culture have primarily stemmed from studies that compare results across various countries, neglecting to investigate culture’s effect on other dimensions. Further research is warranted to determine whether culture moderates conformity and which specific dimensions of culture are implicated, or if factors such as norms contribute to the observed differences.

This systematic review assesses recent studies on conformity and sheds light on a relatively neglected aspect: new research practices (e.g., open data, pre-registration), and ethics. Out of the 48 articles included in the review, only six underwent ethical review by a committee, with four involving minors as participants (Bolderdijk & Cornelissen 2022; Ivanchei et al. 2019; Pham & Buchsbaum 2020; Sibilsky et al. 2021; Yafai et al. 2014; Zhang et al. 2016). The ethical implications of conformity studies are notable, particularly given that researchers often need to deceive participants about the true purpose of the study to observe genuine influence. To align with the Ethical Principles of Psychologists and the American Psychological Association Code of Conduct (APA Code of Ethics 2016), conformity researchers must adhere to rigorous protocols. This includes obtaining informed consent from participants, either in written or verbal form, before the experiment, and providing comprehensive debriefing afterward. Moreover, data must be fully anonymized and made accessible for replication studies. These steps are essential for upholding the ethical standards integral to the study of conformity.

Among the 41 experimental articles, only 10 provided access to study materials or data (Bolderdijk & Cornelissen 2022; Brügger et al. 2019; Egermann et al. 2013; Garcia et al. 2021; Kim et al. 2019; Kyrlitsias et al. 2020; Pham & Buchsbaum 2020; Qin et al. 2022; Schreuter et al. 2021; Sibilsky et al. 2021). Notably, none of the articles mentioned pre-registration. However, it is plausible that data accessibility and open science practices are increasingly adopted, given the recency of the cited articles. This underscores the critical need for rigorous ethical considerations in a field as socially significant as conformity research. One possible answer to these limitations could be to conduct multi-site distributional studies, holding potential for yielding more robust results.

Several factors influencing susceptibility to conformity have been identified, and replications conducted over recent decades have shed light on this phenomenon. However, conformity remains a subject of ongoing study due to lingering ambiguities. While certain determinants of conformity have received considerable attention (e.g., type of paradigm, type of response, characteristics of the majority), others, such as the influence of psychological needs like the need for uniqueness or need to belong, remain relatively unexplored but may significantly contribute. Additionally, while there is some understanding of why individuals conform, strategies for resisting majority influence remain unclear. One of the most important factors in conformity is whether the majority is unanimous. In the articles included in this review, only one article investigates and mentions a minority effect (Qin et al. 2022). Furthermore, the results of this study suggest that the minority (in this case robot-induced) was successful in distracting the participant from the majority response. This finding is consistent with the work of Moscovici (1980) and his conversion theory, which postulates that under certain conditions individuals can also conform to a minority (Moscovici & Naffrechoux 1969). Moscovici (1980) posits that the motivation to conform or not is a function of two factors: the consistency of the source of influence and the individual’s confidence and attachment to their own judgments (Martin & Hewstone 2012; Mugny 1975). Thus, it is the type of behavior of the source of influence, particularly the consistency of responses, that enables conformity and not dependence on this majority, as suggested by Asch’s (1951) approach. This interpretation of majority/minority influence seems to have been set aside in the articles selected for this review in favor of Asch’s (1951) interpretation. Further work focusing on Moscovici’s proposed interpretation could help to enrich the literature, particularly in online conformity, where behavior type, such as response consistency, could play an important role. Future studies could investigate whether there are effective strategies for resisting conformity. Although conformity is predominantly influenced by external factors, in-depth examinations of inter-individual variables may offer insights into resisting social influence.

## Conclusion

This systematic review offers a comprehensive overview of advancements in conformity research since the 2000s. Over the past two decades, studies have consistently demonstrated the robust nature of conformity. Methodologies for measuring conformity have diversified, particularly with the emergence of digital technology, enabling investigations across various contexts (e.g., online, with real/fictional acquaintances, with robots, or artificial intelligence). Despite the prominence of conformity as a research topic, the literature still lacks a definitive understanding of the underlying factors driving this behavior. Presently, no consensus exists regarding the influence of age, gender, or culture on conformity. The determinants of conformity vary depending on specific situational contexts within studies.

Consequently, future research should aim to provide more precise insights into the processes operating under specific contextual conditions, as well as the object of conformity (e.g., moral dilemmas, musical preferences, logic or visual perception tasks). Further studies, including intercultural investigations, meta-analyses, and replications, are necessary to expand and refine our understanding of this multifaceted phenomenon.

## Footnotes

This article is not included in our systematic review as it was published after the review was realized, on 29 November 2023.

Franzen and Mader (2023) found that only openness had an effect, while the other measured traits, such as intelligence, self-esteem, and need for social approval, did not.
